# Enlarged perivascular spaces in multiple sclerosis on magnetic resonance imaging: a systematic review and meta-analysis

**DOI:** 10.1007/s00415-020-09971-5

**Published:** 2020-06-13

**Authors:** Tobias Granberg, Thomas Moridi, Judith S. Brand, Susanne Neumann, Martin Hlavica, Fredrik Piehl, Benjamin V. Ineichen

**Affiliations:** 1 Department of Clinical Neuroscience, Karolinska Institutet, Stockholm, Sweden; 2 Department of Neuroradiology, Karolinska University Hospital, Stockholm, Sweden; 3 Clinical Epidemiology and Biostatistics, School of Medical Sciences, Örebro University, 70185 Örebro, Sweden; 4 Department of Neurosurgery, Inselspital, University of Bern, Bern, Switzerland; 5 Center of Neurology, Academic Specialist Center, Stockholm Health Services, Stockholm, Sweden

**Keywords:** Multiple sclerosis, Enlarged perivascular spaces, Systematic review, Meta-analysis, Magnetic resonance imaging, Biomarker

## Abstract

**Background:**

Perivascular spaces can become detectable on magnetic resonance imaging (MRI) upon enlargement, referred to as enlarged perivascular spaces (EPVS) or Virchow-Robin spaces. EPVS have been linked to small vessel disease. Some studies have also indicated an association of EPVS to neuroinflammation and/or neurodegeneration. However, there is conflicting evidence with regards to their potential as a clinically relevant imaging biomarker in multiple sclerosis (MS).

**Methods:**

To perform a systematic review and meta-analysis of EPVS as visualized by MRI in MS. Nine out of 299 original studies addressing EPVS in humans using MRI were eligible for the systematic review and meta-analysis including a total of 457 MS patients and 352 control subjects.

**Results:**

In MS, EPVS have been associated with cognitive decline, contrast-enhancing MRI lesions, and brain atrophy. Yet, these associations were not consistent between studies. The meta-analysis revealed that MS patients have greater EPVS prevalence (odds ratio = 4.61, 95% CI = [1.84; 11.60], *p* = 0.001) as well as higher EPVS counts (standardized mean difference [SMD] = 0.46, 95% CI = [0.26; 0.67], *p* < 0.001) and larger volumes (SMD = 0.88, 95% CI = [0.19; 1.56], *p* = 0.01) compared to controls.

**Conclusions:**

Available literature suggests a higher EPVS burden in MS patients compared to controls. The association of EPVS to neuroinflammatory or -degenerative pathology in MS remains inconsistent. Thus, there is currently insufficient evidence supporting EPVS as diagnostic and/or prognostic marker in MS. In order to benefit future comparisons of studies, we propose recommendations on EPVS assessment standardization in MS. PROSPERO No: CRD42019133946.

**Electronic supplementary material:**

The online version of this article (10.1007/s00415-020-09971-5) contains supplementary material, which is available to authorized users.

## Introduction

Perivascular spaces surround blood vessel walls penetrating the brain parenchyma through the subarachnoid space and are mostly microscopical [43]. Perivascular spaces seem to play a potential role in the pathogenesis of neuroinflammatory diseases such as multiple sclerosis (MS), as supported by several lines of evidence: perivascular spaces are inhabited by MHC-II presenting macrophages and dendritic cells, as well as patrolling lymphocytes (including T cells) [14, 42]. Perivascular spaces have also been proposed as lymphatic efflux pathways from the brain [29], which is devoid of lymphatic vessels and has long been considered as “immune-privileged” organ [13]. Thus, perivascular spaces seem to represent a hot spot of immune cell interaction. This has also been corroborated using in vivo microscopy in rodents upon initiation of neuroinflammation [4].

Upon a certain size, perivascular spaces can become detectable on magnetic resonance imaging (MRI), referred to as enlarged perivascular spaces (EPVS) or Virchow-Robin spaces [45]. EPVS are detectable on T2- and on T1-weighted MR images as cerebrospinal fluid (CSF)-isointense structures which are correlated with perforating brain vessels [5]. Of note, the exact anatomical compartment of EPVS including their relation to the vascular tree is controversial [12, 16, 32].

Although few scattered perivascular spaces are an almost ubiquitous imaging finding, an increase in EPVS burden has been associated to small vessel disease [3], which increases the risk for stroke, dementia [10], and other neurodegenerative diseases [19, 39].

Accumulating evidence also suggests an association of EPVS to MS. However, as of yet there is no established role for EPVS as imaging biomarker in MS, since existing data is partly contradictory. Thus, some studies describe an association of EPVS with certain disease characteristics, others contradict these findings. Furthermore, studies show considerable methodological differences. The aim of this study was to perform a systematic review and meta-analysis on the current literature on EPVS in MS, including their correlation to other imaging features and clinical characteristics.

## Methods

We registered the study protocol in the International prospective register of systematic reviews (PROSPERO, CRD42019133946, https://www.crd.york.ac.uk/PROSPERO/) and used the Preferred Reporting Items for Systematic Reviews and Meta-Analysis (PRISMA) Guidelines for reporting [34].

### Search strategy

We searched for original observational studies published in full up to 26th of April 2020 in PubMed, Web of Science, and Ovid EMBASE using search terms for EPVS in conjunction with MS/neuroinflammation. See Supplementary Methods for the exact search string in each of these data bases.

### Inclusion and exclusion criteria

We included publications that reported on any outcome related to EPVS, as assessed by MRI, and their associations with any disease feature of MS in humans. Case reports were also included to the systematic review. Exclusions: animal studies, non-English articles, reviews and papers which did not include quantitative data or papers which reported on quantitative data that were previously reported.

### Study selection and data extraction

Titles and abstracts of studies were screened for their relevance in the web-based application Rayyan by two reviewers followed by full-text screening [36]. For more detailed information on data extraction, see supplementary material.

### Quality assessment

The quality of each study was assessed against pre-defined criteria by two reviewers (Supplementary Tables 1 and 2) using QUADAS-2, the revised version of QUADAS [46].

### Data synthesis and analysis

For the meta-analysis, we used summary-level data only. As primary outcome, we assessed differences in EPVS burden between MS patients and controls. Since absolute differences in EPVS were assessed using different methods across studies, standardized mean differences (SMDs) with 95% confidence interval (CI) were reported as measure of association for continuous outcome measures. We also extracted odds ratios (ORs) with 95% CI as relative measure of association for those studies comparing the existence of EPVS in MS patients versus controls. Analyses comparing the burden of EPVS in MS patients vs. controls were stratified by unit of measurement and brain region of EPVS assessment. Secondary outcomes of our meta-analysis included disease-specific associations of EPVS with clinical and neuroimaging biomarkers in MS patients. These associations were assessed using either mean differences or ORs as described above.

For each association of interest, between-study heterogeneity was assessed using the *I*^2^ statistics [22]. SMDs and ORs were pooled using random effects models. A two-tailed *p* value < 0.05 was considered statistically significant. All analyses were carried out using Review Manager Software Version 5.3 (Cochrane, Oxford).

### Publication bias

Publication bias was not assessed since only nine studies were included in the meta-analysis (in the pre-defined protocol, ten studies had been defined as threshold for assessing publication bias).

## Results

### Eligible publications

**Fig. 1:**
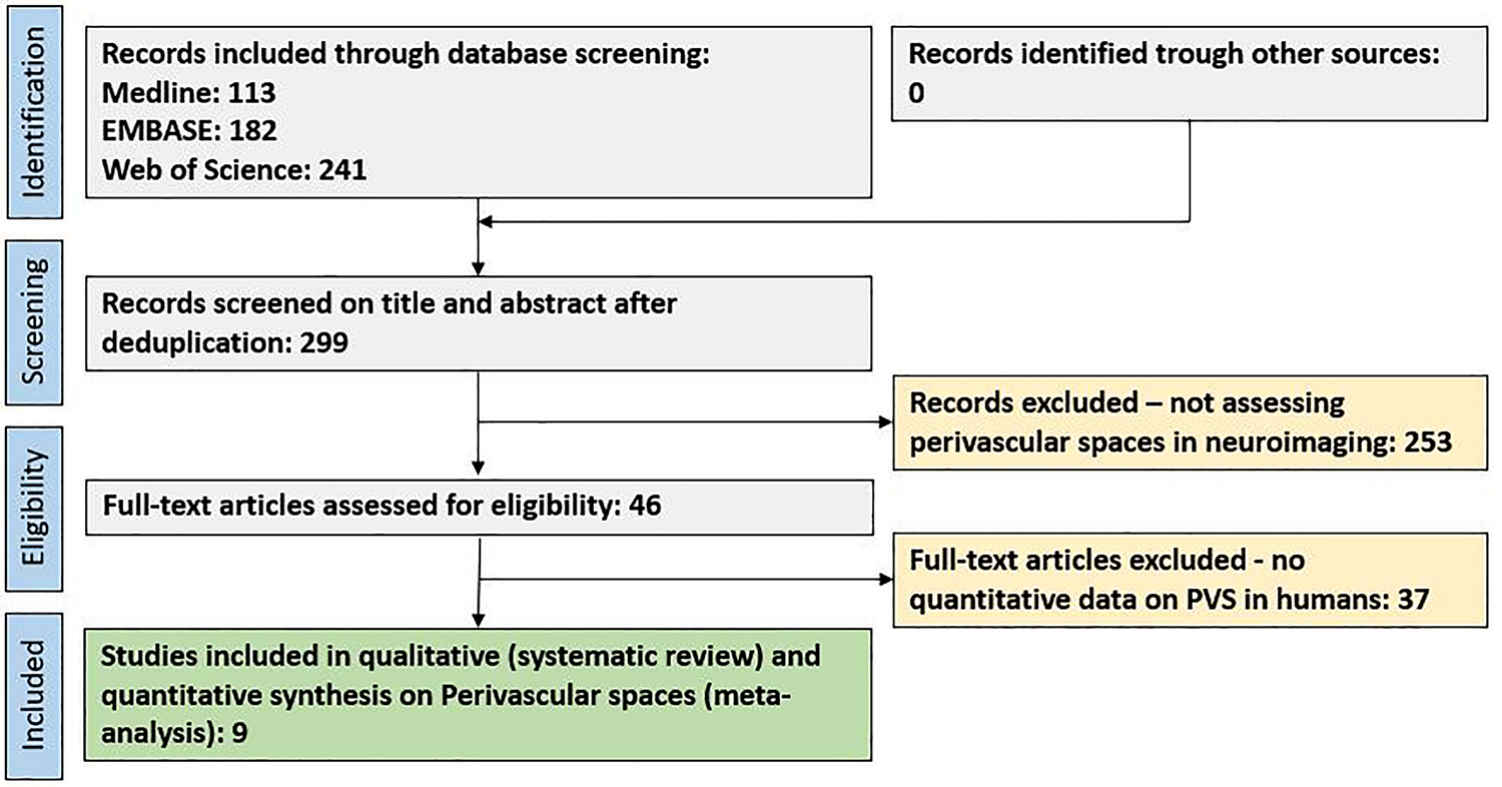
Flow chart depicting the study selection process

In total, 299 original publications were retrieved from our comprehensive data base search. After abstract and title screening, 46 studies were eligible for full-text search. After screening the full text of these studies, nine articles were included for quantitative synthesis (Fig. 1).

### General study characteristics

When pooled, the studies contained a total of 457 MS patients and 352 control subjects. All clinical MS subtypes were represented in the studies (relapsing–remitting MS [RRMS], secondary progressive MS [SPMS], primary progressive MS [PPMS]). One study also included 21 patients with clinically isolated syndrome (CIS) [17]. Table 1 summarizes the included studies assessing the role of EPVS in MS using MRI.

**Table 1:** Summary of study methodology on perivascular spaces (PVS) in multiple sclerosis (MS), in chronological order

First author, year	Data collection and study design	MR imaging: field strength; acquired sequences	*N* (mean age, %female); EDSS; disease duration	Level of adjustment	Criterium	Method of EPVS assessment
Achiron (2002) [1]	Retrospective case–control study	2 T T1: Sagittal, axial, 3 mm T2: Coronal, axial, 3 mm Gd: +	Newly diagnosed MS patients: 71 (26.8, 66%) HC: 60 (27.2, 63%)	Age- and sex-matched controls	Definition	EPVS as punctuate and CSF-isointense on T1 and T2, < 2 mm in diameter, conforming to the path of penetrating arteries, no mass effect
					Region	High convexity
					Raters	2
					Scoring	Grade 0, no EPVS; grade 1, fewer than four EPVS; grade 2, four to seven EPVS; and grade 3, more than seven EPVS [21]
Wuerfel (2008) [48]	Prospective cohort study	1.5 T T1: 3D, 1 mm^3^ T2: Axial, 3 mm T2 FLAIR: Axial, 3 mm Gd: +	RRMS: 45 (39.8, 51%) (18 patients longitudinally) HC: 30 (37.8, 53%) Mean EDSS: 2.3 Mean disease duration: 8.8 years	Age- and sex-matched controls	Definition	EPVS as punctuate and CSF-isointense on FLAIR and T2, conforming to the path of penetrating arteries on MPRAGE. EPVS within MS lesions excluded
					Region	Whole brain
					Raters	2
					Scoring	Manual assessment of EPVS counts and threshold-based semi-automatic post-processing routine in the MedX3.4.3 software package [33, 37, 47] of EPVS counts and volumes
Etemadifar (2011) [15]	Retrospective case–control study	1.5 T T1: Sagittal, axial, 3 mm T2: Sagittal, axial, 3 mm T2 FLAIR: Sagittal, axial, 3 mm Gd: −	Newly diagnosed MS patients: 73 (32.3, 75.3%) HC: 73 (33.3, 75.3%)	Age- and sex-matched controls	Definition	EPVS as punctuate and CSF-isointense on T1, T2 and FLAIR, conforming to the path of penetrating arteries, no mass effect
					Region	High convexity, along lenticulostriate arteries, midbrain
					Raters	2
					Scoring	EPVS counts, shape (oval, round, curvilinear) and diameter (> or < 2 mm)
Al-Saeed (2012) [2]	Retrospective case–control study	1.5 T T1: Coronal, axial T2: Axial T2 FLAIR: Sagittal, axial Gd: +	Newly diagnosed MS patients: 80 Controls with headache: 80	Age- and sex-matched controls	Definition	EPVS as punctuate and CSF-isointense on T1, T2 and FLAIR, conforming to the path of penetrating arteries, no mass effect
					Region	High convexity, along lenticulostriate arteries, midbrain
					Raters	N/A
					Scoring	Grade 0, no EPVS; grade 1, fewer than four EPVS; grade 2, four to seven EPVS; and grade 3, more than seven EPVS [21]. Dilated vs. non-dilated (> or < 2 mm)
Conforti (2014) [9]	Retrospective case–control study	3 T T1: 3D, 1.2 * 1.2 * 1 mm T2: Axial, 3 mm T2 FLAIR: Axial, 3 mm FSPGR T1: 1.2 * 1, 2 * 1 mm Gd: −	MS: 40 (42.7, 70%) (inactive disease) HC: 30 (42.8, 57%) EDSS range: 1–6.5	Age-, sex- and education-matched controls	Definition	EPVS as punctuate and CSF-isointense on T1, T2 and FLAIR, clearly visible on FSPGR, conforming to the path of penetrating arteries, no mass effect
					Region	Whole brain; focus on high convexity, along lenticulostriate arteries, midbrain
					Raters	2
					Scoring	EPVS counts, area and volume – manually circumscribed with semi-automatic delination of the contours using MIPAV
Kilsdonk (2015) [25]	Retrospective case–control study	7 T T1: 3D, 0.49 * 0.49 * 0.4 mm T2 FLAIR: 3D, 0.49 * 0.49 * 0.4 mm Gd: −	RRMS: 22 SPMS: 5 PPMS: 7 (MS: 43, 65%) HC: 11 (38.8, 45%) Mean EDSS: 4 Mean disease duration: 9.4 years	Age- and sex-matched controls	Definition	EPVS as dot-like and CSF-isointense on T1, conforming to the path of penetrating arteries
					Region	a) Handknob, including EPVS in the vertex; b) crus anterius, at its widest; c) anterior commissure; and d) transition between third ventricle and aqueduct, including EPVS in the basal ganglia, and e) peduncles, at the largest interpeduncular distance, including EPVS in the midbrain
					Raters	1 (supervised)
					Scoring	EPVS counts, area and largest cross-section manually delineated on axial MPRAGE using MIPAV. Longitudinal EPVS (running in plane) were excluded
Conforti (2016) [8]	Retrospective case–control study	3 T T1: 3D, 1.2 * 1.2 * 1 mm T2: Axial, 3 mm T2 FLAIR: Axial, 3 mm FSPGR T1: 1.2 * 1, 2 * 1 mm Gd: −	RRMS with Fatigue: 82 (39.08, 70%) HC: 43 (39.63, 63%)	Age-, sex- and education-matched controls	Definition	EPVS as punctuate and CSF-isointense on T1, T2 and FLAIR, clearly visible on FSPGR, conforming to the path of penetrating arteries, no mass effect
					Region	Whole brain; focus on high convexity, along lenticulostriate arteries, midbrain
					Raters	2
					Scoring	EPVS counts, area and volume – manually circumscribed with semi-automatic delineation of the contours using MIPAV
Favaretto (2017) [17]	Retrospective case–control study	3 T T1: 3D, 1 mm^3^ T2 FLAIR: 3D, 1 mm^3^ PSIR: 1 * 1 * 3 mm Gd: +	CIS: 21 (36.3, 76%) RRMS: 15 (35.3, 47%) Progressive MS: 7 (41.3, 57%) HC: 10 (33.0, 60%) Mean EDSS_RRMS_: 2.2 Mean EDSS_PMS_: 6 Mean disease duration_RRMS_: 8.7 years Mean disease duration_PMS_: 13.9 years	Age- and sex-matched controls. Patients without cardiovascular risk factors	Definition	EPVS as punctuate and CSF-isointense on T1, FLAIR and PSIR, conforming to the path of penetrating arteries
					Region	Whole brain
					Raters	3
					Scoring	EPVS counts and volume by manual segmentation in ITK-SNAP. EPVS enlargement (> 2 mm or > 2 mm^3^)
Cavallari (2018) [7]	Retrospective case–control study (from prospective cohort)	1.5 T T2: Axial, 3 mm Gd: +	10-year follow-up: MS patients with disease-worsening: 30 (median 50, 80%) MS patients without disease-worsening: 30 (48, 80%) HC: 15	Age-, sex-, ethnicity-, disease duration- and EDSS-matched controls (patients with vs. without disease-worsening). Multivariate analysis adjusted for age, sex, disease duration and baseline EDSS	Definition	Small, sharply-delineated structured with CSF-isointensity measuring < 3 mm in cross-sectional diameter; following the course of perforating vessels [36]
					Region	High convexity, along lenticulostriate arteries, midbrain
					Raters	1
					Scoring	Counts and Potter rating scale: 0 = none, 1 = 1–10 EPVS, 2 = 11–20 EPVS, 3 = 21–40 EPVS, 4 = > 40 EPVS per region [36]

*EDSS* expanded disability status scale, *EPVS* enlarged perivascular spaces, *FLAIR* fluid-attenuated inversion recovery, *FSPGR* fast spoiled gradient echo, *Gd* gadolinium, *HC* healthy control, *MPRAGE* magnetization-prepared rapid acquisition with gradient echo, *MR* magnetic resonance, *MS* multiple sclerosis, *PD* proton density, *PSIR* phase-sensitive inversion recovery, *PMS* progressive multiple sclerosis, *RRMS* relapsing–remitting multiple sclerosis

Two out of nine studies had a prospective design with a follow-up of up to ten years [7, 48]. Increasing age is a known contributor to the presence of EPVS in the general population [19] and previous studies have shown variations in EPVS between men and women [26]. All studies were at least matched on age and sex. EPVS have also been associated with cerebrocardiovascular disease [19, 39]. One study reported that no patient had cardiovascular risk factors [17]. Table 1 summarizes patient cohorts and methodology of the included studies.

### Methodological assessment of perivascular spaces using magnetic resonance imaging

One major difference between the studies was the difference in the static magnetic field strength of the used MR imaging system: Four studies used 1.5 T (T); one study used 2 T; three studies used 3 T; and one study used 7 T.

**Fig. 2:**
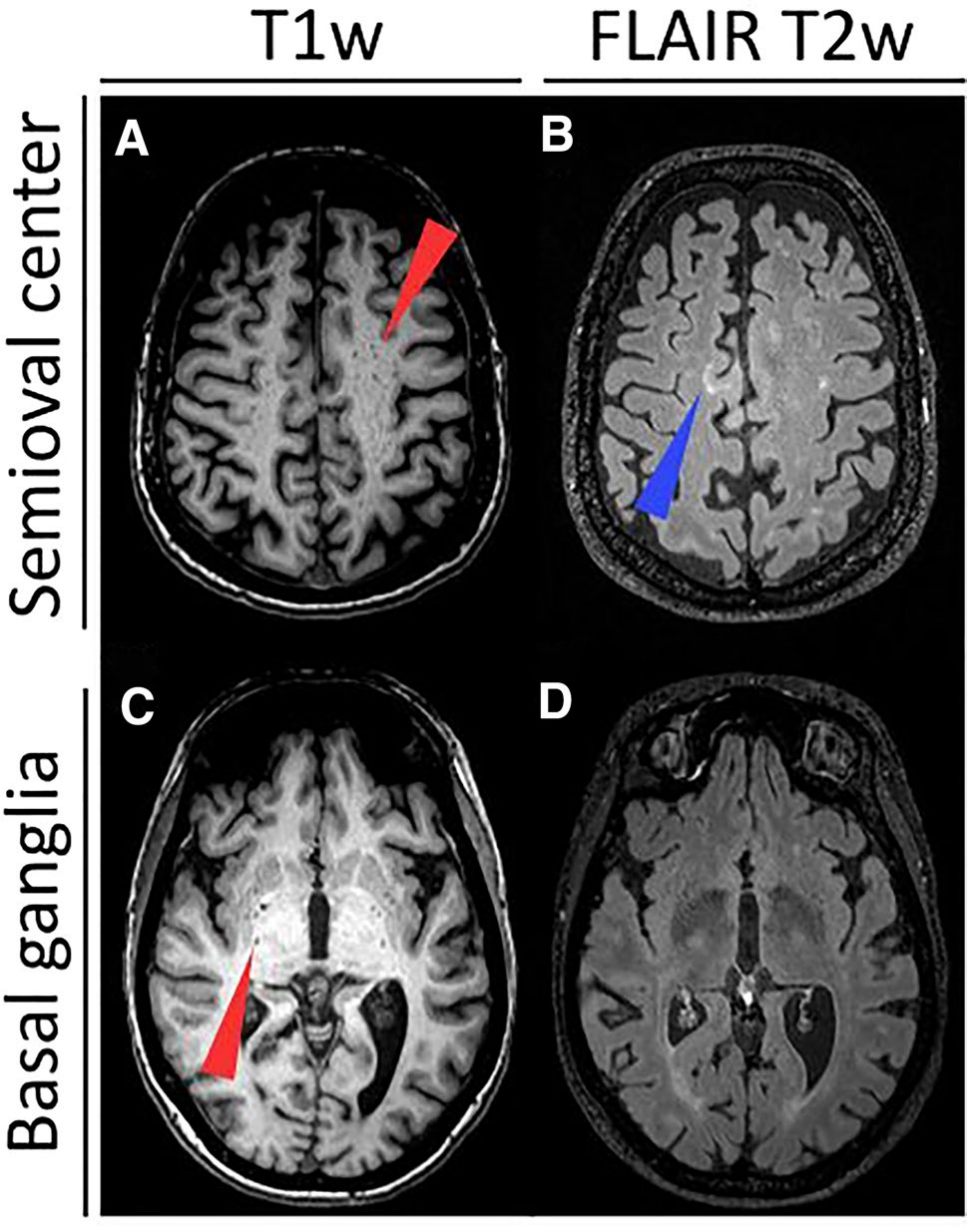
Enlarged perivascular spaces (EPVS) are readily detectable on T1- and fluid-attenuated inversion recovery (FLAIR) T2-weighted (T1w, T2w) magnetic resonance imaging (MRI) as cerebrospinal fluid-isointense punctuate structures (red arrowheads) in the semioval center (**a** and **b**) and in the basal ganglia (**c** and **d**). In contrast, multiple sclerosis lesions appear hyperintense on FLAIR T2-weighted images (blue arrowhead in **b**)

All studies defined EPVS as cerebrospinal fluid (CSF)-isointense punctuate structures on T1- and/or T2-weigted images, as well as their conforming path along penetrating arteries, e.g. in the semioval center or along the lenticulostriate arteries. All except one study assessed EPVS on T1- and T2-weighted images, the remaining study only used T2-weighted images to assess EPVS [7]. Acquired MR sequences per study are listed in Table 1. Most studies further report that EPVS can readily be discriminated from MS lesions or small lacunes by their hypointense appearance on fluid-attenuated inversion recovery [FLAIR] T2-weighted images, in contrast to the hyperintense appearance of MS lesions/lacunes on FLAIR T2-weighted images (Fig. 2). One study used phase-sensitive inversion recovery (PSIR) enabling enhanced T1 contrast [31], which may have resulted in higher sensitivity to detect EPVS [17].

In seven out of nine studies, two or more independent raters quantified EPVS. Interrater agreement was reported by three studies and ranged between 90 and 100% [1, 17, 48]. In two studies, only a single rater quantified EPVS.

While there was a high level of agreement on how to define EPVS, there were considerable differences in how the studies measured EPVS: all studies assessed EPVS count (manually using software packages such as MIPAV or ITK-SNAP or semi-automatic using threshold-based post-processing pipelines [48]) and three studies also used semi-quantitative rating scales for EPVS counts [1, 2, 7], either according to Heier et al. [21] or Potter et al. [38]. In addition to EPVS counts, four studies measured EPVS volume [8, 9, 17, 48]; four studies categorized EPVS as dilated or non-dilated (using a threshold of 2 mm or 2 mm^3^) [2, 8, 15, 17]; three studies measured the EPVS area on representative axial slices [8, 9, 25]; two studies either excluded EPVS > 2 mm [1] or < 2 mm [17]; and one study assessed the shape of EPVS (round, oval or curvilinear) [15].

The anatomical location of EPVS assessment also varied across studies. Three characteristic locations for EPVS assessment have been proposed—along the lenticulostriate arteries, semioval center and brain stem [28]. Four studies assessed EPVS in these locations [2, 7, 8, 15]; one study further subcategorized these three locations into a total of five different locations [25]. Three studies quantified EPVS in the whole brain without reporting area-specific-counts [9, 17, 48] and one study only assessed EPVS in the semioval center [1].

### Differences in enlarged perivascular spaces comparing MS patients to controls

**Fig. 3:**
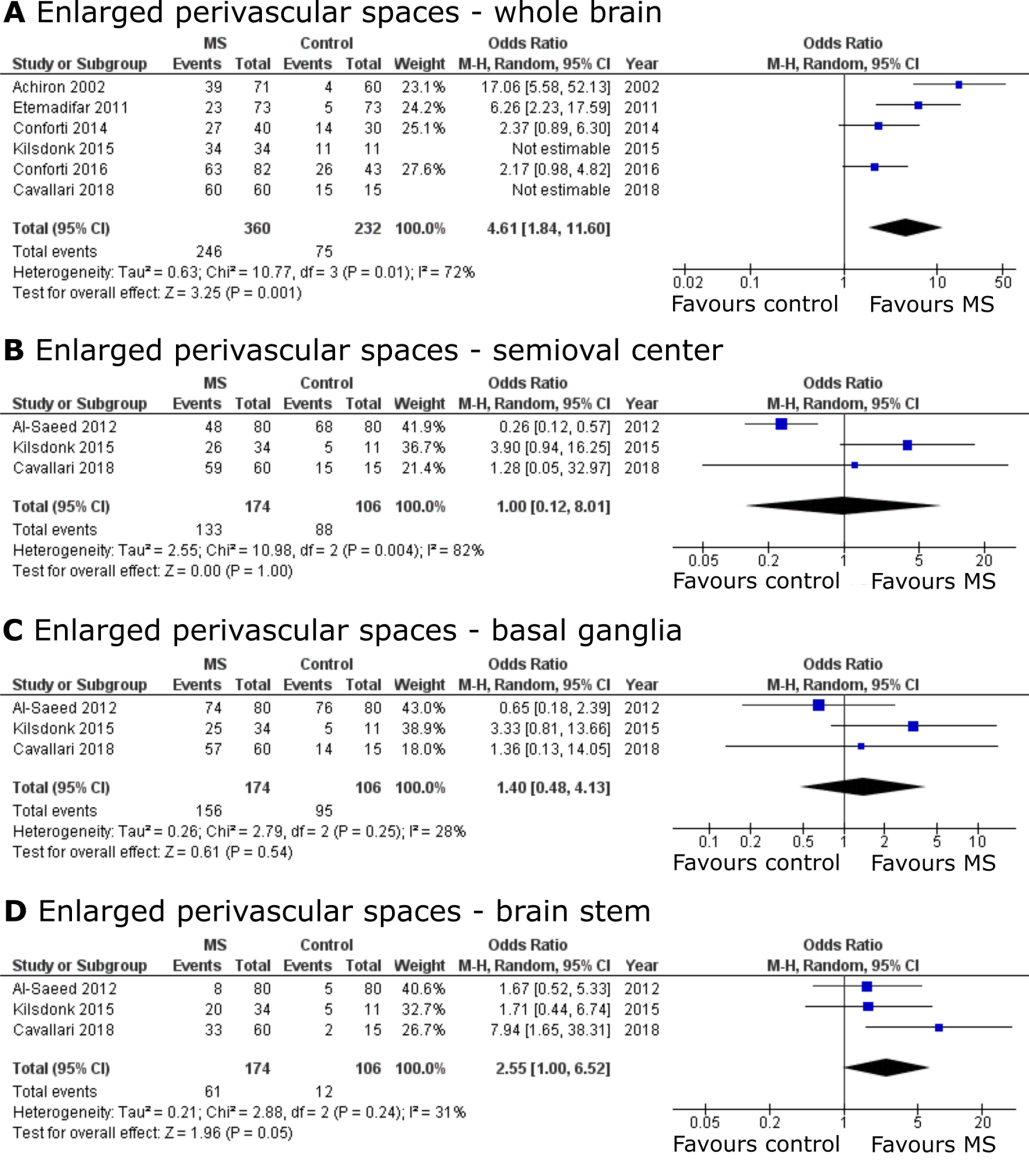
Pooled analyses of studies comparing the odds of enlarged perivascular spaces (EPVS) in multiple sclerosis (MS patients versus controls. Odds ratios (ORs) for EPVS comparing MS patients to controls were extracted and pooled using the random effects Mantel–Haenszel method. Pooled analyses were stratified by brain region of EPVS assessment: **a** whole brain, **b** semioval center, **c** basal ganglia and **d** brain stem. Of note, odds ratios were not estimatable in two studies assessing whole brain EPVS as EPVS in these studies were detected in all MS patients and controls [7, 25]. *CI* confidence interval, *df* degrees of freedom, *M–H* Mantel–Haenszel

We retrieved ORs for whole brain EPVS from six studies comparing MS patients to controls (including a total of 360 MS patients and 232 controls). Data from two studies could not be included in our meta-analysis due to non-estimatable ORs [7, 25]. According to our meta-analysis, whole brain EPVS were more common in MS patients than in controls (OR 4.61, 95% CI [1.84; 11.60], *p* = 0.001, Fig. 3a). There was, however, substantial heterogeneity between studies assessing whole brain EPVS (*I*^2^ = 72%) [23]. In stratified analyses by brain region a similar trend of association between EPVS and MS was found for studies assessing EPVS in the brain stem (*p* = 0.054, Fig. 3d), whereas no strong evidence of an association was observed in studies assessing EPVS in the semioval center (Fig. 3b) and basal ganglia (Fig. 3c). Also, between-study heterogeneity was substantial among studies assessing EPVS in the semioval center.

**Fig. 4:**
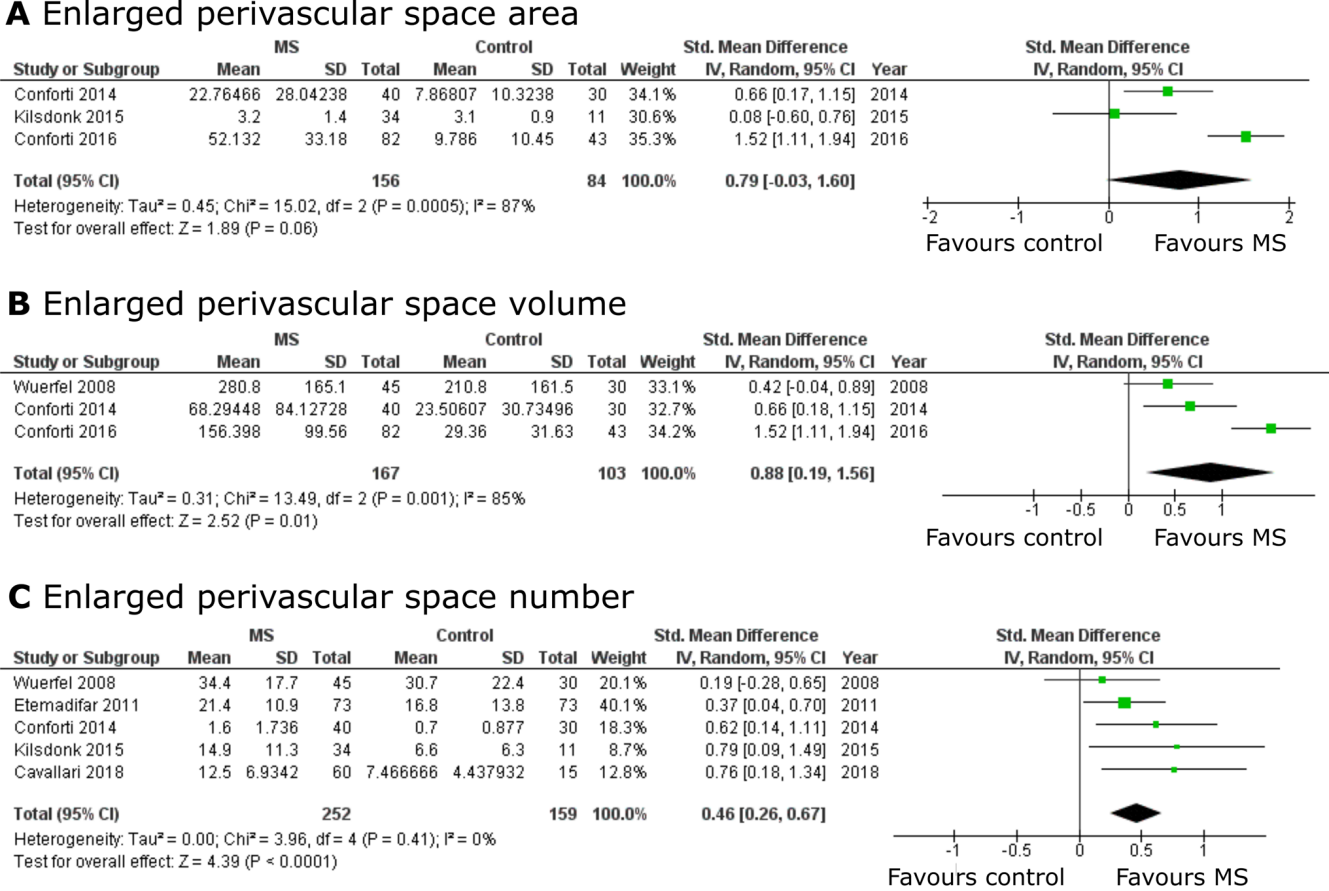
Pooled analyses of studies assessing absolute differences in enlarged perivascular spaces (EPVS) comparing MS patients to controls. Standardized mean differences were pooled using the random effects inverse-variance weighted method. Pooled analyses were stratified by unit of measurement: **a** EPVS area, **b** EPVS volume and **c** EPVS count. *CI* confidence interval, *df* degrees of freedom, *IV* inverse variance weighted

A total of six studies assessed absolute differences in EPVS outcome measures between MS patients and controls. Results of these analyses, stratified by unit of measurement (i.e. area, volume, count) are summarized in Fig. 4. Overall, a similar pattern of association was observed for each unit of measurement, with SMDs not being different in these analyses, even though the SMD for EPVS area was only borderline significant. I.e. compared to controls, MS patients had a larger EPVS volume (SMD = 0.88, 95% CI = [0.19; 1.56]), area (SMD = 0.79, 95% CI = [− 0.03; 1.60]) and count (SMD = 0.46, 95% CI = [0.26; 0.67]), with the latter being consistent with the whole brain ORs observed.

Studies examining associations of EPVS with MRI brain volume or lesion measures did not yield sufficient numbers for meta-analysis, and these results are therefore summarized individually below.

### Associations of enlarged perivascular spaces with clinical and imaging determinants in MS patients

#### Age

Two studies investigated the contribution of ageing to EPVS in MS: one study found an increase in EPVS counts in MS patients with increasing age [25] whereas the other only found such an association in the control group [48].

#### Sex

One study reported that male sex is a positive contributor to EPVS numbers in the semioval center in MS patients [15]. No such difference was observed in the control group.

#### Clinical outcomes

Five studies assessed the association between EPVS and physical disability, measured either by expanded disability status scale (EDSS) [9, 17, 25, 48] or general neurological disability [1]—none of these found an association of EPVS with physical disability. One study found a positive trend (*p* = 0.051) between EPVS number and fatigue [8], as measured by the fatigue severity scale (FSS) [27]. This analysis was, however, not adjusted for age, sex or MS disease features. Another study found a positive correlation between dilated EPVS (> 2 mm) and cognitive decline in MS [17], again without adjustment for potential confounding factors.

#### Imaging outcomes

Studies assessing the association of EPVS with gadolinium (Gd) contrast-enhancing MRI lesions are conflicting. The first study investigating the association with Gd-enhancing MRI lesions in MS patients was by Achiron and Faibel [1]. In their retrospective study consisting of 71 newly diagnosed MS patients, no association between EPVS counts and Gd-enhancing lesions was observed. Another retrospective study including 43 MS patients corroborated these findings showing no association between EPVS count and volume with Gd-enhancing lesions [17]. In contrast, however, a prospective study including 45 MS patients, with longitudinal follow-up of 18 MS patients for 12 months found a larger EPVS volume and a higher EPVS counts accompanying the occurrence of new Gd-enhancing lesions [48]. Yet, four studies consistently report the absence of an association between EPVS counts/volumes and T2 lesions [17, 25], two of which used a prospective design [7, 48].

The association of EPVS with brain atrophy has been assessed in six out of nine studies. Five out of six studies did not find an association between EPVS and brain volumetry [7–9, 17, 48]. In all these studies, brain volumes have been computed using the package SIENAX from the software FSL [24]. One study including 34 MS patients and 11 healthy controls did, however, detect an association between EPVS and supratentorial brain fraction as computed by the surface-based software FreeSurfer [18]. Notably, it is the only study employing supratentorial brain fraction as outcome. All other studies used whole brain parenchymal fraction (BPF) [40], defined as the ratio of total brain parenchymal volume to the total intracranial volume, as the volumetric outcome. Moreover, the study by Kilsdonk and colleagues employed by far the highest MRI field strength (7 T) for the image acquisition and should thus have a very high EPVS detection sensitivity [25].

## Discussion

### Main findings

In the nine studies included in our systematic review, we found considerable methodological differences between the studies hampering direct comparisons. In particular, this involved the MRI acquisition (B_0_ field strength, imaging protocol) and the methodology for EPVS quantification (location, EPVS count vs. volume vs. area). Nevertheless, our meta-analysis of these nine studies showed that overall EPVS are more frequently observed in MS patients than in control subjects. Moreover, MS patients display higher EPVS counts and larger EPVS volumes compared to control subjects.

### Findings in the context of existing evidence

The role of EPVS in the MS pathophysiology remains poorly elucidated, in part due to inconsistent evidence. Of note, the exact anatomical definition of EPVS is also still a matter of debate [12]. This includes the content of EPVS: the scarce available evidence assessing the location of EPVS within the vascular tree indicates a correlation of EPVS with arterioles but not venules [5, 6]. However, this needs further elicudation. While EPVS are not specific for MS, our meta-analysis indicates that MS patients have higher EPVS volumes and counts compared to controls. The higher prevalence of EPVS in MS patients could hint towards disturbances in CNS fluid drainage and/or excess fluid leakage from the vasculature [35, 44]. However, it should be noted, that given the design of most of the studies included in this meta-analysis (i.e. mostly case control studies with retrospective data collection), the temporality of the association with EPVS presence and size could not reliably be addressed. Hence, it is unclear whether EPVS burden is a potential risk factor for MS, or merely represents an epiphenomenon of the disease process. From a clinical point of view, findings from this review suggest a potential prognostic value of EPVS. However, since the independent predictive value and discriminatory power of EPVS has not been addressed in studies to date, this requires further investigation.

Notably, the only study reporting an association of EPVS counts to brain atrophy used the highest applied MRI field strength (7 T) and estimated supratentorial brain fraction via the software FreeSurfer [25] in contrast to studies which were not able to reproduce this association [7–9, 17, 48]. It has been shown previously that these mentioned factors can contribute considerably to differences in brain volumetry measures [20, 30]. From a pathophysiological viewpoint, the enlargement of perivascular spaces in MS (and other neurodegenerative disorders) could represent a local ex vacuo dilatation.

The association of EPVS to Gd-enhancing lesions is another matter of debate: one study with a prospective design demonstrated an association of EPVS volume and number to contrast-enhancing lesions thereby establishing EPVS as marker for ongoing neuroinflammation [48]. The authors speculated that local infiltration of immune cells as a perivascular cuff may lead to an enlargement of individual EPVS. Two studies did, however, not confirm these findings [1], only one associating EPVS volume (and not number) to contrast-enhancing lesions though [17].

The included studies used similar criteria to define EPVS, namely CSF-isointensity on T1- and T2-weighted images without mass effect. Nevertheless, the studies used a plethora of different EPVS characteristics and clinical/imaging outcome measures. EPVS outcome measures included semiquantitative rating scales [21, 38], counts, volumes, areas, diameters, or shapes. Some studies also only included EPVS above or below a certain diameter (2 mm as most commonly used cut-off). Similarly, a variety of different methods to assess imaging outcomes including brain atrophy or lesion measures were used by the studies (manual segmentation, semi-automated or fully automated approaches).

## Limitations

Our study has a few limitations: (1) for most outcomes, only few studies could be included in our meta-analysis. This reflects the substantial methodological differences between the studies. (2) The presence of substantial heterogeneity between studies precluded the ability to explore sources of this heterogeneity using subgroup analyses. (3) For EPVS frequency between MS patients and control subjects, the OR was not estimatable in two studies in which EPVS were apparent in all patients and controls. Thus, the association of EPVS with MS might have been overestimated when pooling ORs; however, results for differences in EPVS count (which is an equivalent outcome measure) were similar to those observed for ORs, corroborating the presence of a positive association between EPVS and MS. (4) EPVS have consistently been associated to cerebrovascular disease and neurodegeneration [19, 39]. Yet, only one of the included studies reported that patients had no cardiovascular risk factors [17]. Nevertheless, the mean age of the MS patient cohorts was presumably prior to relevant cerebrocardiovascular and neurodegenerative disease onset.

In order to better define the role of EPVS as imaging biomarker, more studies with prospective design are required to minimize potential bias, which is apparent in some of the included studies. Standardization of EPVS measurement, not least to emend comparability between studies, is another requirement.

### Recommendations

To foster reliability and comparability of future studies, we recommend the following routine for EPVS imaging in MS: (1) the imaging protocol should include both a T1- and T2-weighted sequence, (2) at least 2 raters should independently assess EPVS, and (3) EPVS should not only be quantified in the whole brain, but also in different brain regions (semioval center, basal ganglia and brain stem). It is also noteworthy that several promising automated methods to quantify EPVS have been proposed whose future application will further increase reliability and comparability of studies [11, 41]. Finally, assessing the shape of EPVS should also be considered since different EPVS characteristics might reflect different underlying pathologies. Of note, the EPVS characteristics which define its role as imaging biomarker does not necessarily need to be similar for different diseases, e.g. between primary neurodegenerative and neuroinflammatory disorders such as MS—in MS, measuring the diameter of EPVS might better reflect a local accumulation of immune cells whereas increasing EPVS numbers might better reflect an ongoing widespread ex vacuo atrophy in cognitive decline.

## Conclusions

This meta-analysis is the first to report at an aggregated level that the prevalence of EPVS is higher in MS patients, with higher EPVS burden compared to controls. This supports a potential role of EPVS in MS etiopathogenesis and its use as marker with prognostic potential. The role of EPVS as disease severity biomarker remains uncertain, however, not least to considerable methodological differences between studies as well as the limited number of studies addressing the role of EPVS as potential prognosticator. Standardization of EPVS assessment will improve future comparability between studies. Finally, studies on correlation between MRI EPVS and underlying histopathology could offer valuable clues on the role of EPVS in MS.

## Electronic supplementary material

Below is the link to the electronic supplementary material.

Supplementary methods (DOCX 13 kb)

Supplementary search string (DOCX 13 kb)

Supplementary tables (DOCX 27 kb)

## Data Availability

Data available from the authors upon request.
