# Supplementary material for: Enlarged perivascular spaces in multiple sclerosis on magnetic resonance imaging: a systematic review and meta-analysis
Source: J Neurol. 2020 Jun 13;267(11):3199–212. doi: 10.1007/s00415-020-09971-5 (PMC7577911; doi:10.1007/s00415-020-09971-5)
Supplement: Supplementary file 1 — Supplementary methods (DOCX 13 kb) [file 415_2020_9971_MOESM1_ESM.docx]

**Supplementary methods**

Two reviewers independently extracted title, authors, publication year, study design, number of subjects per group, MS subtypes, mean age, sex, EPVS location, all EPVS measures, method of EPVS assessment and unit of measurement as well as technical imaging parameters from each included study.

Any results of associations, including if analyses were adjusted for confounders or not were extracted. Effect estimates adjusted for the largest number of confounders were extracted when studies reported more than one effect estimate for the association of interest. Discrepancies in findings were resolved by discussion among assessors. In case means or measures of variance were missing for certain outcomes or if no quantitative data were reported for specific outcomes, corresponding authors were contacted by email to ensure complete data for analysis. We contacted 4 authors for missing data of whom 3 provided additional data for the meta-analysis.
