# Supplementary material for: Enlarged perivascular spaces in multiple sclerosis on magnetic resonance imaging: a systematic review and meta-analysis
Source: J Neurol. 2020 Jun 13;267(11):3199–212. doi: 10.1007/s00415-020-09971-5 (PMC7577911; doi:10.1007/s00415-020-09971-5)
Supplement: Supplementary file 2 — Supplementary search string (DOCX 13 kb) [file 415_2020_9971_MOESM2_ESM.docx]

**Supplementary search string**

1.) Medline:

((exp Multiple sclerosis/) OR (encephalomyelitis disseminata or multiple sclerosis).ti,ab,kf.) AND ((Glymphatic System/) OR (virchow robin space* or perivascular space* or paravascular space* or paravascular system* or glymphatic* or meningeal lymphatic vessel*).ti,ab,kf.)

Limited to English language

2.) Embase

(('multiple sclerosis'/mj) OR ( ‘encephalomyelitis disseminata':ti,ab,kw OR 'multiple sclerosis':ti,ab,kw)) AND (('glymphatic system'/de) OR ('virchow robin space*':ti,ab,kw OR 'perivascular space*':ti,ab,kw OR 'paravascular space*':ti,ab,kw OR 'paravascular system*':ti,ab,kw OR glymphatic*:ti,ab,kw OR 'meningeal lymphatic vessel*':ti,ab,kw))

AND [English]lim

3.) Web of Science

TOPIC: (("encephalomyelitis disseminata" or "multiple sclerosis")

AND

TOPIC: (("virchow robin space*" or "perivascular space*" or "paravascular space*" or "paravascular system*" or glymphatic* or "meningeal lymphatic vessel*"))

Refined by: LANGUAGES: ( ENGLISH )
