# Supplementary material for: Enlarged perivascular spaces in multiple sclerosis on magnetic resonance imaging: a systematic review and meta-analysis
Source: J Neurol. 2020 Jun 13;267(11):3199–212. doi: 10.1007/s00415-020-09971-5 (PMC7577911; doi:10.1007/s00415-020-09971-5)
Supplement: Supplementary file 3 — Supplementary tables (DOCX 27 kb) [file 415_2020_9971_MOESM3_ESM.docx]

| **Domain** |  | **Question** | **Comment** |
| --- | --- | --- | --- |
| Patient selection | Risk of bias | Was a consecutive or random sample of patients enrolled? | **Nothing mentioned = unclear** |
|  |  | Did the study avoid inappropriate exclusions? | **Nothing mentioned = unclear** |
|  |  | **Could the selection of patients have introduced bias?** |  |
|  | Applicability | **Is there concern that the included patients do not match the review question?** | **e.g. by having outdated diagnostic criteria for MS, such as Poser criteria or McDonalds 2012 (or earlier revisions of the McDonalds criteria)** |
| Index Test | Risk of bias | Were the assessors blinded for the groups during the EPVS scoring? |  |
|  |  | Did at least 2 readers assess EPVS scoring? |  |
|  |  | **Could the conduct or interpretation of the MRI have introduced bias?** |  |
|  | Applicability | **Is there concern that the MRI, its conduct, or interpretation differ from the review question?** | **Are the MRI pulse sequences likely at demonstrating EPVS at a sufficient resolution?** |
| Reference standard | Risk of bias | Is the conducted MRI likely to correctly classify EPVS? | **Is there at least a T1 and T2 sequence in the MRI protocol?** |
|  |  | **Could the MRI, its conduct, or its interpretation have introduced bias in detection of EPVS?** |  |
|  | Applicability | **Is there concern that EPVS as defined by the MRI does not match the review question?** | **e.g. by not defining EPVS as CSF-isointense punctuate structures or not excluding DDs in other pulse sequences (such as MS lesions which are hyperintense in T2 compared to EPVS)** |
| Flow and timing | Risk of bias | Was there an appropriate interval between MRI and clinical outcome? | up to 6 months for a clinical/cognitive assessment is considered as appropriate interval |
|  |  | Did all patients underwent the same MRI scanning procedure? | Differences in scanning procedures for patients, eg. different sequences and field strengths |
|  |  | Were all patients included in the analysis? | Same patient count included to the statistical analysis as initially included in the study |
|  |  | **Could the patient flow have introduced bias?** |  |

**Supplementary table 1**: Predefined QUADAS-2 criteria [1].

| **Domain** | **Sub-domain** | **Achiron 2002** | **Wuerfel 2008** | **Etemadifar 2011** | **Al-Saeed 2012** | **Conforti 2014** | **Kilsdonk 2015** | **Conforti 2016** | **Favaretto 2017** | **Cavallari 2018** |
| --- | --- | --- | --- | --- | --- | --- | --- | --- | --- | --- |
| **Patient selection** | Risk of bias | Low risk | Low risk | Low risk | Low risk | High risk | Low risk | Unclear | High risk | Low risk |
|  | Applicability | High risk | High risk | High risk | High risk | High risk | High risk | High risk | High risk | High risk |
| **Index Test** | Risk of bias | Unclear risk | Low risk | Unclear risk | Unclear risk | Low risk | High risk | Low risk | Unclear | High risk |
|  | Applicability | Low risk | Low risk | Low risk | Low risk | Low risk | Low risk | Low risk | Low risk | Low risk |
| **Reference standard** | Risk of bias | Low risk | Low risk | Low risk | Low risk | Low risk | Low risk | Low risk | Low risk | High risk |
|  | Applicability | Low risk | Low risk | Low risk | Low risk | Low risk | Low risk | Low risk | Low risk | Low risk |
| **Flow and timing** | Risk of bias | Low risk | Low risk | Low risk | Low risk | Low risk | Low risk | Low risk | Low risk | High risk |

**Supplementary table 2**: Risk of bias using the pre-defined QUADAS-2 criteria [1].

**References**

[1] P.F. Whiting, A.W. Rutjes, M.E. Westwood, S. Mallett, J.J. Deeks, J.B. Reitsma, M.M. Leeflang, J.A. Sterne, and P.M. Bossuyt, QUADAS-2: a revised tool for the quality assessment of diagnostic accuracy studies. Annals of internal medicine 155 (2011) 529-36.

[2] A. Achiron, and M. Faibel, Sandlike appearance of Virchow-Robin spaces in early multiple sclerosis: a novel neuroradiologic marker. AJNR. American journal of neuroradiology 23 (2002) 376-80.

[3] J. Wuerfel, M. Haertle, H. Waiczies, E. Tysiak, I. Bechmann, K.D. Wernecke, F. Zipp, and F. Paul, Perivascular spaces--MRI marker of inflammatory activity in the brain? Brain : a journal of neurology 131 (2008) 2332-40.

[4] M. Etemadifar, A. Hekmatnia, N. Tayari, M. Kazemi, A. Ghazavi, M. Akbari, and A.H. Maghzi, Features of Virchow-Robin spaces in newly diagnosed multiple sclerosis patients. European journal of radiology 80 (2011) e104-8.

[5] O. Al-Saeed, R. Athyal, M. Ismail, and M. Sheikh, Significance of Virchow-Robin spaces in patients newly diagnosed with multiple sclerosis: a case-control study in an Arab population. Medical principles and practice : international journal of the Kuwait University, Health Science Centre 21 (2012) 447-51.

[6] R. Conforti, M. Cirillo, P.P. Saturnino, A. Gallo, R. Sacco, A. Negro, A. Paccone, G. Caiazzo, A. Bisecco, S. Bonavita, and S. Cirillo, Dilated Virchow-Robin spaces and multiple sclerosis: 3 T magnetic resonance study. Radiol Med 119 (2014) 408-14.

[7] I.D. Kilsdonk, M.D. Steenwijk, P.J. Pouwels, J.J. Zwanenburg, F. Visser, P.R. Luijten, J. Geurts, F. Barkhof, and M.P. Wattjes, Perivascular spaces in MS patients at 7 Tesla MRI: a marker of neurodegeneration? Multiple sclerosis (Houndmills, Basingstoke, England) 21 (2015) 155-62.

[8] R. Conforti, M. Cirillo, A. Sardaro, G. Caiazzo, A. Negro, A. Paccone, R. Sacco, M. Sparaco, A. Gallo, L. Lavorgna, G. Tedeschi, and S. Cirillo, Dilated perivascular spaces and fatigue: is there a link? Magnetic resonance retrospective 3Tesla study. Neuroradiology 58 (2016) 859-66.

[9] A. Favaretto, A. Lazzarotto, A. Riccardi, S. Pravato, M. Margoni, F. Causin, M.G. Anglani, D. Seppi, D. Poggiali, and P. Gallo, Enlarged Virchow Robin spaces associate with cognitive decline in multiple sclerosis. PloS one 12 (2017) e0185626.

[10] M. Cavallari, S. Egorova, B.C. Healy, M. Palotai, J.C. Prieto, M. Polgar-Turcsanyi, S. Tauhid, M. Anderson, B. Glanz, T. Chitnis, and C.R.G. Guttmann, Evaluating the Association between Enlarged Perivascular Spaces and Disease Worsening in Multiple Sclerosis. Journal of neuroimaging : official journal of the American Society of Neuroimaging 28 (2018) 273-277.
